# Half-Breed Micro-Organisms

**Published:** 1883-10

**Authors:** C. M. Wright

**Affiliations:** Cincinnati


### Half Breed Micro-Organisms.

BY PROF. C. M. WRIGHT, CINCINNATI.

Several of my friends have become convinced from much that has been said in Dental Societies, and much that has been written on the subject, that the micro-organisms found in the mouth and in cavities of decay in human teeth are *animal* and not *vegetable* forms of life. Believing this to be an error, or at least not in accordance with the *generally accepted* views of advanced modern students of these low forms of life, I beg leave to say that, though the line of demarkation between the animal and vegetable kingdoms has been shifted about a great deal, and is not very distinct at any time, yet for certain reasons, the forms of life that we have been interested in—the bacteria, etc., are now classified by most observers as belonging on the *vegetable* side of the line. It was once believed that living action could be exhibited only by organized structure, but now we know that all the functions of life may be carried on by minute "jelly-specks," in whose apparently homogeneous, semi-fluid substance, nothing like organization can be detected. We know also that in the highest organisms the *essential* part of life-work is done by the same material that is found in the "jelly-speck." The higher organism is only the mechanism, the structure through which this substance exerts its wonderful properties. Huxley has called this protoplasm, "The Physical Basis of Life."

The microscope has made us acquainted with a multitude of minute and simple forms of life, which are essentially particles of protoplasm, each kind having usually a definite shape and size, and showing to the careful observer, some distinctive character or habits of life as we learn more about them; how they grow; how they multiply; where they thrive, etc., we learn from their habits of life, to distinguish them as vegetable or animal in character. We can classify them as living plants and living animals, as we can plants, and animals that do not require the microscope for their observation. The distinction is very fine sometimes, we must admit, as in these low forms of life, the line as we have said is not so distinct; cannot be, from the nature of the case, and especially, as the forms, whose life history interest us, partake, to a certain extent, of some of the attributes of animal, as well as vegetable, life. They might almost be called *half-breeds*, from their life on the border land between the vegetable and animal kingdoms. By selecting a cell, or simple form, and cultivating it, a complete life-history can be made out. The bacteria, in common with many others, have received this special study, and have been set down (by some authors) as belonging to the fungi. Five varieties or rather types have been recognized. 1, micrococcus; 2, bacterium; 3, bacillus; 4, vibriones; 5, spirillum. These are very low or simple forms of the group of fungi, and perhaps the simplest type of vegetation representing the simple cell. They belong to the protophytic fungi, and the group has been named the Schiro-mycetes. Beale calls this a distinct class from the fungi, but acknowledges its vegetable character. They thrive best in the midst of decomposing or decomposable organic matter. They appear to relish and utilize such products. They are aquatic in their habits, and show some varieties according to the media in which they live. They differ from green plants, in not being able to form carbon compounds by the decomposition of carbonic acid, and requiring for their support carbo-hydrates or their derivatives. They resemble ordinary plants, however, by the power they possess of forming out of ammonia, *nitrogenous compounds*, which animals cannot do.—Carpenter. They consist of minute cells. They multiply by sub-division. Most of them have the power, at least at some stage of their existence, of swimming about, more or less rapidly in the liquid they inhabit. Tyndall and others have shown that the bacteria, bacilli, etc., have wonderful powers of resisting cold; and certain degrees of warmth or heat. Their power of spontaneous motion, and their appearance in the field of the microscope have, perhaps, caused the belief in their animal character, though *motion* is not distinctive of animal life—witness the desmids and diatoms. Perhaps the clearest reason for their classification among the vegetables is the character of their *nutrition.* This may, perhaps, be taken as the most generally applicable characteristic upon which the classification of these border-land organisms, that most closely approximate to one another, may be made. If we find a low form of life depending for its nourishment upon *organic compounds already formed,* which, in some way or other it takes into its own body, we admit that this, though not an *absolute,* is yet a *general* characteristic of the animal kingdom. On the other hand, if our little cell possesses the power of *producing* the *organic compounds* at the expense of *inorganic elements,* with which it is supplied by air and water, and applies these for the increase of its own fabric, we acknowledge that this is a prominent attribute of the *vegetable world.* If the vegetable had not this power, what would become of animal life? There are, however, certain plants which resemble animals in their dependence upon organic compounds prepared by other organisms. There are plants that are unable themselves "to effect that fixation of carbon, by the decomposition of carbonic acid of the atmosphere, which is the stage in their production." This is the case among the entire group of *fungi* though, however, the fungi seem generally to depend rather upon organic matter in a state of deoomposition—many of them having the power of *promoting* by fermentation, that process.

Then there are some animals in whose tissues starch, cellulose, and chlorophyl are found. This is a characteristic of *vegetables.* It has not, I believe, been proved that the animals, in whose tissue these organic substances have been found, really *generate* these compounds for themselves, by the decomposition of carbonic acid.

From studies of the life histories, and the nutrition of these lower forms of life, our micrococci, bacteria, bacilli, etc., have, from their effect upon the balance in this tiny scale, been set down on the *vegetable side* of the delicate and wavy line of demarkation. They are in English, "funguses,"—"*microscopic fungi.*" Modern scientific writers accept the classification of the observers. It is, perhaps, only the popular idea, that they are "bugs" and have teeth, or gimlets, or augers, capable of boring into the hard enamel, etc. It is, perhaps, only the popular dental idea, that enamel rods may become Aaron rods, and turn into serpents before the lenses of the miscroscopist, or that the canaliculi are harbors for immense fleets of aquatic battering rams that will not rest in haven, but must batter down the quays and produce general destruction. *Theories* in regard to pathological conditions of the teeth built upon such uncertain foundations cannot stand, and are only confusing obstacles in the pathway of science. Elementary studies in the botany and zoology of the miscroscopic Garden of Eden should *precede* the erection of pathological hypothesis.

Permit me to quote two of the conclusions, Nos. 5 and 6, from Dr. W. D. Miller's paper—one of the very best, by the way, that has ever graced our dental periodical literature on this subject, and is worthy of the careful attention of every student—the quotation proves that Dr. Miller, in January, 1883, accepted the classification as *fungi* of those micro-organisms.

"5. The fungi produce anatomical and pathological changes in the deep layers, stop up the canaliculi and necessarily lead, sooner or later, to the death of the dentinal fibrils. The outer layers of dentine, thereby deprived of nourishment, die and fall a prey to putrefactive agents.

"6. The invasion of the fungi is always preceded by the extraction of the lime salts."

There are a very large number of vegetable organisms of simple structure found in the human body, in health and disease, on the surfaces, in the fluids, in internal tissues and organs, into which the germs must have previously penetrated. Wherever animal or vegetable matter (be it healthy or diseased) is undergoing disintegration and decay, low organisms grow and multiply. Lionel S. Beale, in "The Microscope in Medicine," says of the mode in which fungi gain entrance into the system of living beings: "Vegetable organisms, found even in the substance of the inmost tissues, result from the development of germs introduced from without. Since we know that the germs of many *entozoa* make their way very readily through the tissues of the organism, there is no difficulty in explaining how bodies so very much smaller than these, *as the germs of that fungi*, are introduced. These germs have the power of insinuating themselves through the firmest tissue, and multiply in number as they make their way through."

He believes "that there is reason to think that often they possess individual powers of growth and multiplication, *long before they have grown large enough for us to see them*, even with the aid of the highest magnifying power we possess."

In referring in the same work to the question of this paper, Dr. Beale says, on page 498: "Even the vegetable nature of fungi has been called in question by some, but it would be absurd to reply to those who entertain such utterly untenable opinions."

Bilroth says: "Foul air is always associated with the development of minute elementary organisms belonging to the *lowest vegetable species, microscopic fungi, and algae.* They are sometimes minute globules (micrococcus), sometimes minute rods (bacteria), etc.

"In putrefaction they play the same part as the *ferment fungus* does in the fermentation of the juice of the grape, and many other fruits; they are the so-called ferments which induce putrefaction of the juices of the body."

Prof. Cassidy says in a late paper: "Bacteria cannot construct protein, from the mineral kingdom."

I could multiply quotations to an almost indefinite extent, but have probably said enough to call in question, at least, the popular idea of the purely *animal nature* of bacteria, etc. The incredulous student, who does not respect modern authorities, may consider them as *half-breeds*, as we do the mixed races of our Indian borders, in whose blood traces of white and Indian can be found in unequal and varying proportions, and who are surely neither Indians nor whites. The subject of these lower forms of micro-organisms is by no means a settled one. New light may be thrown upon them any day by some of the patient investigators; but it seems to me, that *now* the weight of evidence classifies correctly the bacteria, as belonging to the *garden* and *not* the *menagerie.*—*Ohio State Journal of Dental Science.*

